# The Negative Influence of High-Glucose Ambience on Neurogenesis in Developing Quail Embryos

**DOI:** 10.1371/journal.pone.0066646

**Published:** 2013-06-20

**Authors:** Yao Chen, Jian-xia Fan, Zhao-long Zhang, Guang Wang, Xin Cheng, Manli Chuai, Kenneth Ka Ho Lee, Xuesong Yang

**Affiliations:** 1 Key Laboratory for Regenerative Medicine of the Ministry of Education, Division of Histology and Embryology, Medical College, Jinan University, Guangzhou, China; 2 Department of Gynecology and Obstetrics, International Peace Maternity and Child Health Hospital Affiliated to Shanghai Jiaotong University School of Medicine, Shanghai, China; 3 Division of Cell and Developmental Biology, University of Dundee, Dundee, United Kingdom; 4 Stem Cell and Regeneration Thematic Research Programme, School of Biomedical Sciences, Chinese University of Hong Kong, Shatin, Hong Kong; 5 Institute of Fetal-Preterm Labor Medicine, Jinan University, Guangzhou, China; State Key Laboratory of Reproductive Biology, Institute of Zoology, Chinese Academy of Sciences, China

## Abstract

Gestational diabetes is defined as glucose intolerance during pregnancy and it is presented as high blood glucose levels during the onset pregnancy. This condition has an adverse impact on fetal development but the mechanism involved is still not fully understood. In this study, we investigated the effects of high glucose on the developing quail embryo, especially its impact on the development of the nervous system. We established that high glucose altered the central nervous system mophologically, such that neural tube defects (NTDs) developed. In addition, we found that high glucose impaired nerve differentiation at dorsal root ganglia and in the developing limb buds, as revealed by neurofilament (NF) immunofluorescent staining. The dorsal root ganglia are normally derived from neural crest cells (NCCs), so we examine the delamination of NCCs from dorsal side of the neural tube. We established that high glucose was detrimental to the NCCs, *in vivo* and *in vitro*. High glucose also negatively affected neural differentiation by reducing the number and length of neurites emanating from neurons in culture. We established that high glucose exposure caused an increase in reactive oxidative species (ROS) generation by primary cultured neurons. We hypothesized that excess ROS was the factor responsible for impairing neuron development and differentiation. We provided evidence for our hypothesis by showing that the addition of vitamin C (a powerful antioxidant) could rescue the damaging effects of high glucose on cultured neurons.

## Introduction

Gestational diabetes is diagnosed as high blood sugar levels initiated at pregnancy. Its emergence is principally attributed to reduced insulin function by pregnant hormones during pregnancy. Women with this condition generally show no obvious symptoms and it is not life threatening. The blood glucose level in these individuals usually drops back to normal after delivery. Nevertheless, diabetes during pregnancy does have an adverse impact on both the mother and the developing embryo. It has been reported that maternal hyperglycemia leaded to 4–12.9% deformities of the cardiovascular system and central nervous system (CNS). Other important organs in the fetus are also correspondingly affected and fetal mortality is up to 50% [1], [2], [3]. High levels of glucose in pregnant woman reaches the fetus through the placenta, which not only directly damages the myocardial cells but also causes excessive apoptosis [4]. In rat embryos, high glucose increases the incidence rate of NTDs, reduces somite numbers and crown-rump length [5]. In shell-less chick embryo culture system, it has been shown that glucose exposure at 50 mM and 100 mM resulted in a embryo mortality rate of over 70% and produced a variety of malformations ranging from growth retardation to abnormal heart development [6]. Elevated glucose in pregnant women could also lead to a significant increase in emacrosomia, presentation anomalies, polyhydramnios, preeclampsia and gestational hypertension [7]. In this context, it is important to fully understand how gestational diabetes affects embryonic development and the mechanisms involved, in order to develop new therapies for this condition.

Neurulation is the initial stage in the development of the CNS. During this morphogenetic process the neural folds elevate and fuse together to form the neural tube, which is the precursor of the spinal cord. When these neural folds fail to properly close it leads to NTDs [8], [9]. Another crucial neurulation event is the delamination of the cranial and trunk NCCs formed at the edge of dorsal neural tube. One group of trunk NCCs migrate dorsal-laterally between the ectoderm and somites, and will later differentiate into melanocytes. While a second group of trunk NCCs migrate to eventually form the sympathetic ganglia [10]. In higher vertebrates, the posterior half of each sclerotome repulses the migrating motor axons and the NCCs (precursors of peripheral nervous system (PNS) sensory ganglia) forcing them instead to move preferentially through the anterior sclerotome [11]. This pattern of NCCs migration is believed to lay the foundation for the segmental organization of the neurons [12].

The role of ROS in the pathophysiology of preeclampsia has recent been a topic of great interest [13]. Depending on the nature of the ROS species, they are rapidly detoxified by various cellular enzymatic and non-enzymatic mechanisms [14]. In the retina of human diabetics, acellular capillaries develop and this event is associated with endothelial cell death [15]. It has been claimed that oxidative stress increases in the diabetic retina and that the use of antioxidants could inhibit glucose-induced mitochondrial dysfunction and capillary degeneration [16]. Vitamin C is a recognized water-soluble antioxidant that could protect cellular components from damages induced by ROS [17]. Normally, the transport of vitamin C through the cell membranes is facilitated by glucose transporters, especially GLUT1 [18], [19]. Furthermore, vitamin C is synthesized from glucose in most species and has the ability to scavenge ROS [19], [20]. Vitamin C may function as a chain-breaking antioxidant in the lipid phase by an interacting with lipid-soluble antioxidants such as vitamin E and coenzyme Q [20].

## Results

### Toxic Effect of High Glucose on Quail Embryos

**Figure 1:**
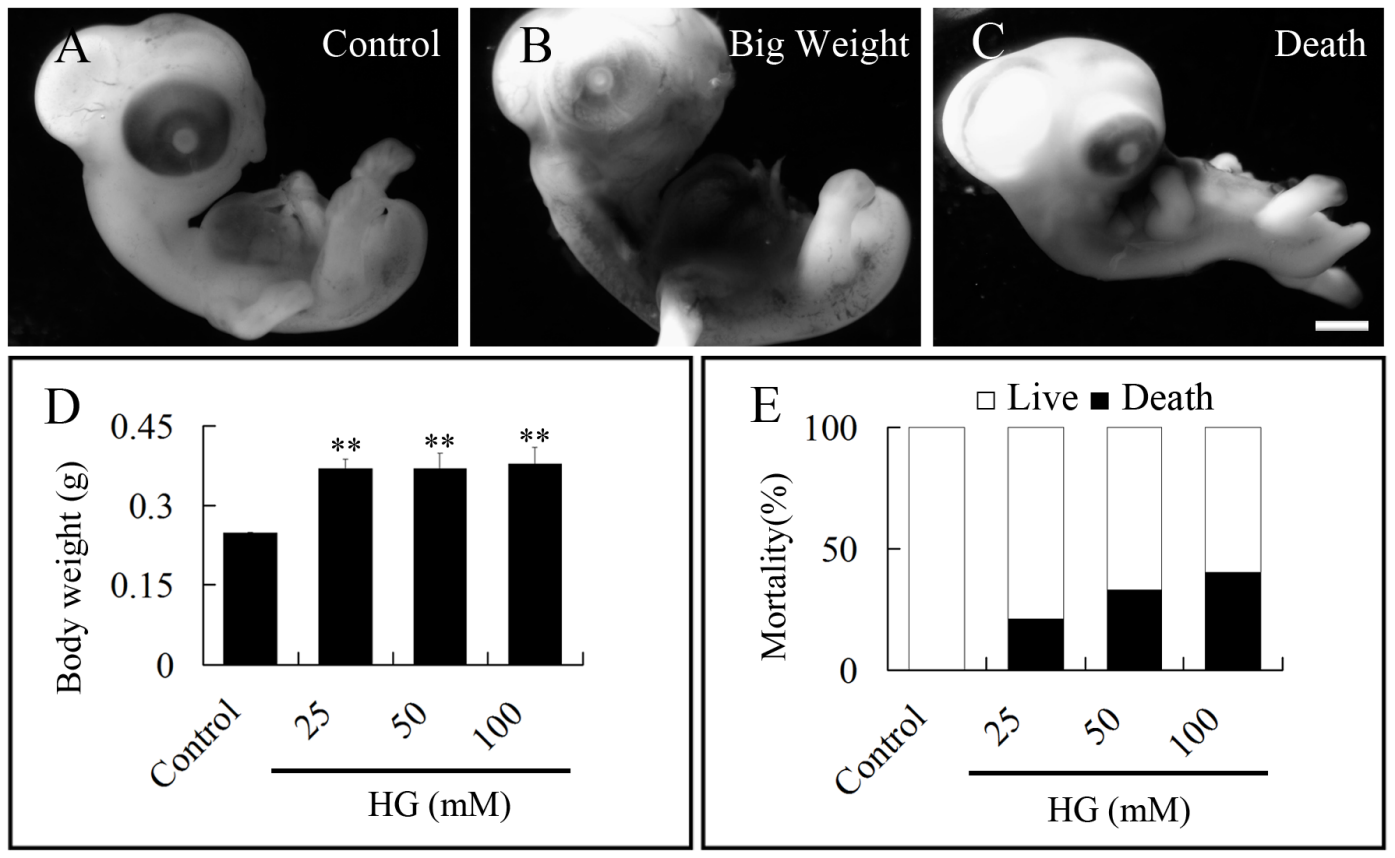
Effects of high glucose on the body weight and mortality of 5.5-day old quail embryos. Fertilized quail eggs were exposed to 0–100 mM glucose solution for 5.5 days and then the embryos were weighed and checked for embryo mortality. **A–C:** Representative appearance of control, enlarged and dead quail embryos following high glucose exposure. **D:** Bar chart showing the weight of quail embryos after glucose treatment (**p<0.01, compared with the control). **E:** Bar chart showing the rate of embryo mortality after high glucose treatment. Abbreviations: HG, high glucose. Scale bar = 1 mm.

It has been reported that maternal hyperglycemia could damage the developing embryo [1]. Hence, we examined the effects of various high dose of glucose on morphogenesis in the quail embryo. The weight of control and glucose-treated embryos were measured and also whether the high glucose caused embryo death was established. We found that exposure to high glucose significantly increased the weight of 5.5-day old quail embryos when compared with control embryos (Figs. 1A and B). The embryos were administered three different high doses of glucose 25, 50 and 100 mM and embryo number = 12, 15, 13, respectively (Fig. 1D). The weight of experimental embryos significantly increased in all three high dose of glucose relative to the control (control embryos: n = 10; p<0.01). However, there was no significant difference in embryo weight amongst the different glucose treatment groups. Dead embryos were not weighed and excluded from calculations. The mortality rate was also determined in 5.5-day old embryos and revealed that embryo death increased with in a dose dependant fashion (Figs. 1C and E).

### Influence of High Glucose on Trunk Neural Tube Development

**Figure 2:**
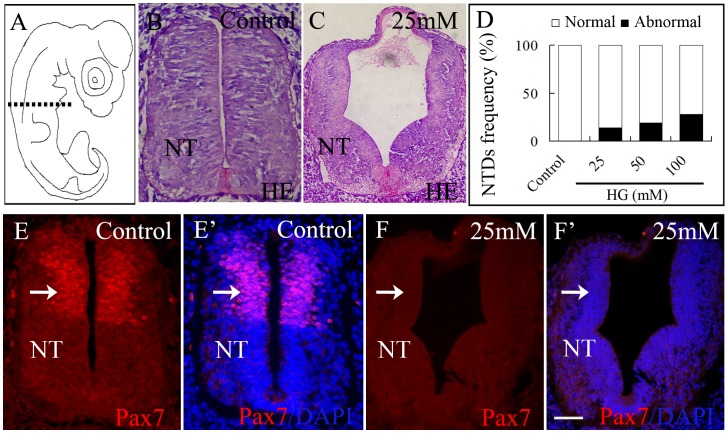
High glucose induces trunk level neural tube dysplasia. **A:** Quail embryos were exposed to high glucose for 3.5 days, sectioned transversely and stained with H & E or immunofluorescently stained for Pax7. **B, C:** showing the representative appearance of the trunk neural tube in control and 25 mM glucose-treated embryos. **D:** Bar chart showing the incidence rate of NTDs increased with increases in glucose exposure. Pax7 is normally expressed in the dorsal side of the neural tube (**E**) but in the presence of high glucose Pax7 expression was inhibited (**F**). **E’, F’:** Sections counterstained with DAPI. Abbreviations: NT, neural tube. Scale bar = 50 µm.

The neural tube is the first structure to emerge during neurulation. Hence, we examined the effect of high glucose on neural tube development. We established that the neural tube of the embryo failed to close properly at 25 mM glucose treatment group. It induced the neural tube to develop abnormally compared with untreated embryos (Figs. 2A–C). The incidence rate for neural tube dysplasia increased with increased concentrations of glucose (Fig. 2D). The Pax7 gene plays a very important role in the process of neural tube closure [21], so we examined how elevated levels of glucose affected expression of this gene. In control embryos, Pax7 (a myogenic regulatory factor) was expressed in the dorsal side of the neural tube (Figs. 2E and E’), dermomyotome and delaminated NCCs. In contrast, in the presence of 25 mM glucose, Pax7 expression was significantly repressed at the dorsal site of the dysplastic neural tube (Figs. 2F and F’).

### Influence of High Glucose on the Dorsal Root Ganglia Development

**Figure 3:**
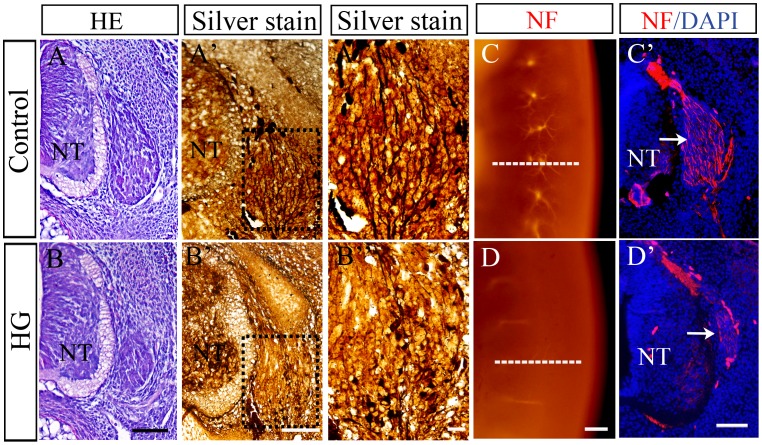
Effects of high glucose on neurons in the dorsal root ganglia of 5.5-day old quail embryos. Transverse sections of the trunk neural tube region of control (A) and high glucose treated (B) embryos. These sections were also **s**ilver stained and revealed that there were fewer **s**ilver^+^ neurons in high glucose treated embryos than control embryos (A’–B”). Furthermore, the sections were stained with NF antibody and confirmed that there were significantly fewer neuron in the high glucose treated embryos (C–D’**).** Abbreviations: NT, neural tube. HG, high glucose. Scale bars = 100 µm in A–B, 50 µm in A’,B’; 10 µm in A”,B”; 200 µm in C,D and 100 µm in C’,D’.

The effects of high glucose on PNS development was examined by immunofluorescent staining for neuron specific intermediate filament (NF) marker and by silver staining (Fig. 3). In control embryos, the dorsal root ganglia appeared large and tear-drop shaped (Fig. 3A). However, the dorsal root ganglia appeared significantly smaller in the high glucose-treated embryos (Fig. 3B). In order to visualize the entire neuron, silver staining was performed on transverse sections of the dorsal root ganglia (Figs. 3A’–B’). In the control embryos (Figs. 3A’–A”), there were significantly more silver-stained nerve fibers than embryos on high glucose (Figs. 3B’–B”). Likewise, in 5.5-day whole mount embryo staining, NF expression at trunk level was weaker in the high glucose treated embryos (Fig. 3D) than in control embryos (Fig. 3C). The corresponding transverse sections showed the distinct differences in NF-labeling of the dorsal root ganglia (Figs. 3C’–D’).

### Inhibitory Effects of High Glucose on the Neural Crest Cells

**Figure 4:**
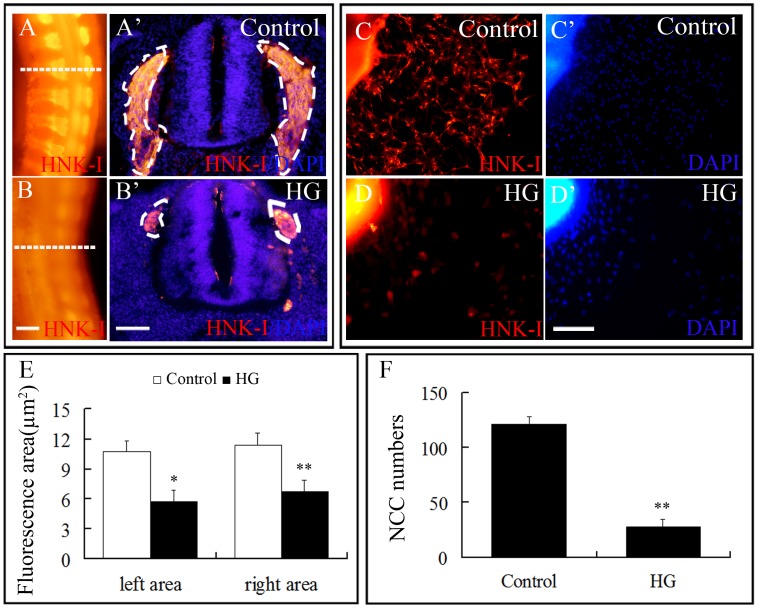
High glucose retards NCC migration. **A–B:** Whole-mount 3.5-day old embryos stained with HNK-1 antibody (NCC marker). **A’–B’:** Transverse sections of A and B showing that under high glucose, NCC migration was dramatically inhibited. **C–D:** Control neural tube explants (C, C’) and explants treated with high glucose (D, D’) were stained with HNK-1 antibody and DAPI dye. The results revealed that high glucose also inhibited NCC migration, *in vitro*. **E:** Bar chart comparing the areas occupied by NCCs in transverse sections of control and high glucose-treated embryos. There was a significant reduction of HNK-1^+^ areas in HG group (*p<0.05, **p<0.01, compared with the control). **F:** Bar chart comparing the number of HNK-1^+^ NCCs between control and high glucose-treated cultures (**p<0.01). Abbreviations: HG, high glucose. Scale bar = 200 µm in A–B, 50 µm in A’–B’, 100 µm in C–D and C’–D’.

We determined that in the presence of high glucose, Pax7 expression was not only inhibited in dorsal side of the neural tube but also in migratory NCCs (Fig. 2F). Therefore, we performed immunostaining for HNK-1 (a specific marker for migratory NCCs) to determine if the high glucose exposure affected the migration of NCCs *in vivo* and *in vitro* (Fig. 4). Some of these cells will contribute to the dorsal root ganglia which belong to the PNS. At trunk level in 3.5-day embryo, HNK-1^+^ cells were evident between the neural tube and somites (Fig. 4A). In the presence of high glucose, there was a significant reduction in HNK-1 expression compared with the control embryos (Fig. 4B), which is more apparent in the transverse sections (Figs. 4A’–B’). Comparisons of the area occupied by HNK-1^+^ cells revealed that there were significantly fewer NCCs following high glucose exposure (Fig. 4E). In tissue culture, neural tube explants revealed that cells would migrate out from the edges of neural tube after 4-hour incubation. Using this *in vitro* NCCs assay, the delamination of NCCs was investigated after high glucose treatment. We found that HNK-1 expression was stronger in control explants (Fig. 4C) than the high glucose treated explants (Fig. 4D). However, there was not much difference in the total number of cells emigrated from between the different explants, as revealed by DAPI staining (Figs. 4C’–D’). In another word, high glucose specifically affected the HNK-1^+^ NCCs (Fig. 4F). In sum, high glucose impaired NCCs development as revealed by our *in vivo* and *in vitro* experiments.

### Influence of High Glucose on the Sensory Nerves of 5.5-dayold Limb Buds

**Figure 5:**
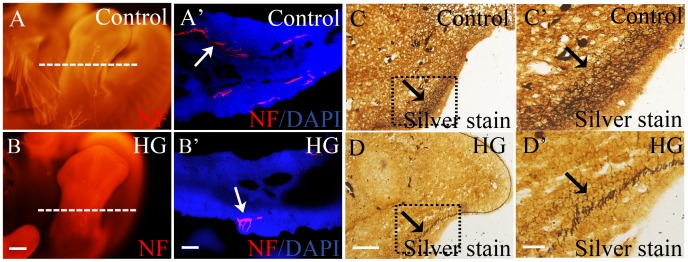
High glucose impaired peripheral nerve development in the limb buds. **A, B:** Whole-mount 5.5-day old embryos were stained with NF antibody or silver following high glucose treatment. **A’, B’:** Transverse sections (white dotted lines in A and B) of the limb buds revealed there were fewer NF^+^ nerve fibers in high glucose-treated embryos than control embryos. **C, C’, D’, D:** Silver staining also confirmed there were fewer nerve fibers in the high glucose-treated embryos than control embryos. Scale bar = 500 µm in A, B; 50 µm in A’, B’, C, D and 20 µm in C’, D’.

We next examined the development of sensory nerves in the developing limb bud as an index to evaluate the influence of high glucose exposure. In 5.5-day old embryos, NF-labeling revealed that there were significantly more NF^+^ nerves present in control (Figs. 5A and A’) than in high glucose treated limb buds (Figs. 5B and B’). This was also confirmed by silver staining of paraffin sections of the limb buds (Figs. 5C–D).

### Cytotoxic Effect of High Glucose on Neuron Development in vitro

**Figure 6:**
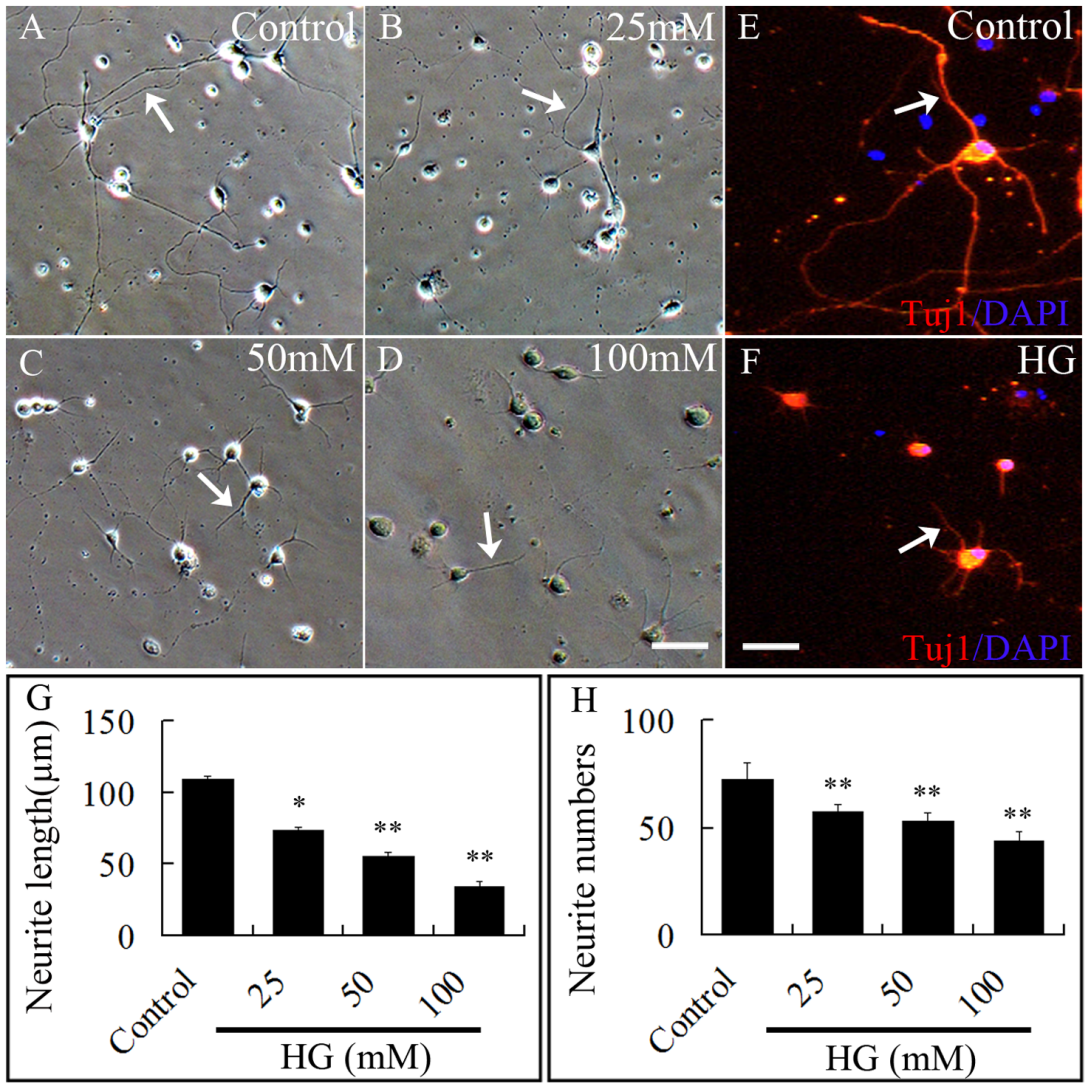
High glucose represses neuron development. Neurons were harvested from the brains of quail embryos and cultured various concentrations of high glucose. Neuron development was evaluated by determining the number and morphology of neurites protruding from the cultured neurons. **A–D:** Morphology of neurons in the presence of 0, 25 mM, 50 mM and 100 mM glucose. The average neurite length was found to be significantly reduced as the concentration of glucose was increased. **E, F:** Tuj1 immunostaining which labels the neurites confirmed high glucose shortens the length of the neurites. **G:** Bar chart showing high glucose significantly shortens the neurites (*p<0.05, **p<0.01, compared with the control). **H:** Bar chart showing the number of neurites protruding from the neurons were also significantly reduced (**p<0.01, compared with the control). Scale bars = 50 µm in A–D and 20 µm in E–F.

We treated primary neuron cultures with 0–100 mM of glucose for 24 hours. The results demonstrated that there was a remarkable difference in neurite length, numbers and neuron diopter amongst control and high glucose treated cultures (Figs. 6A–D). The number of neurons in cultures exposed to 25–100 mM glucose was significantly fewer (Fig. 6G) and length of the neurons shorter (Fig. 6H) than in the control cultures (*p<0.05, **p<0.01). We immunofluorescently stained the neuron cultures with Tuj1 antibody and showed that the glucose treated neurites were significantly shorter (Fig. 6F) than untreated neurons (Fig. 6E).

### High Glucose-induced Impairment of Neuron Development could be Mediated though Excess ROS Generation

**Figure 7:**
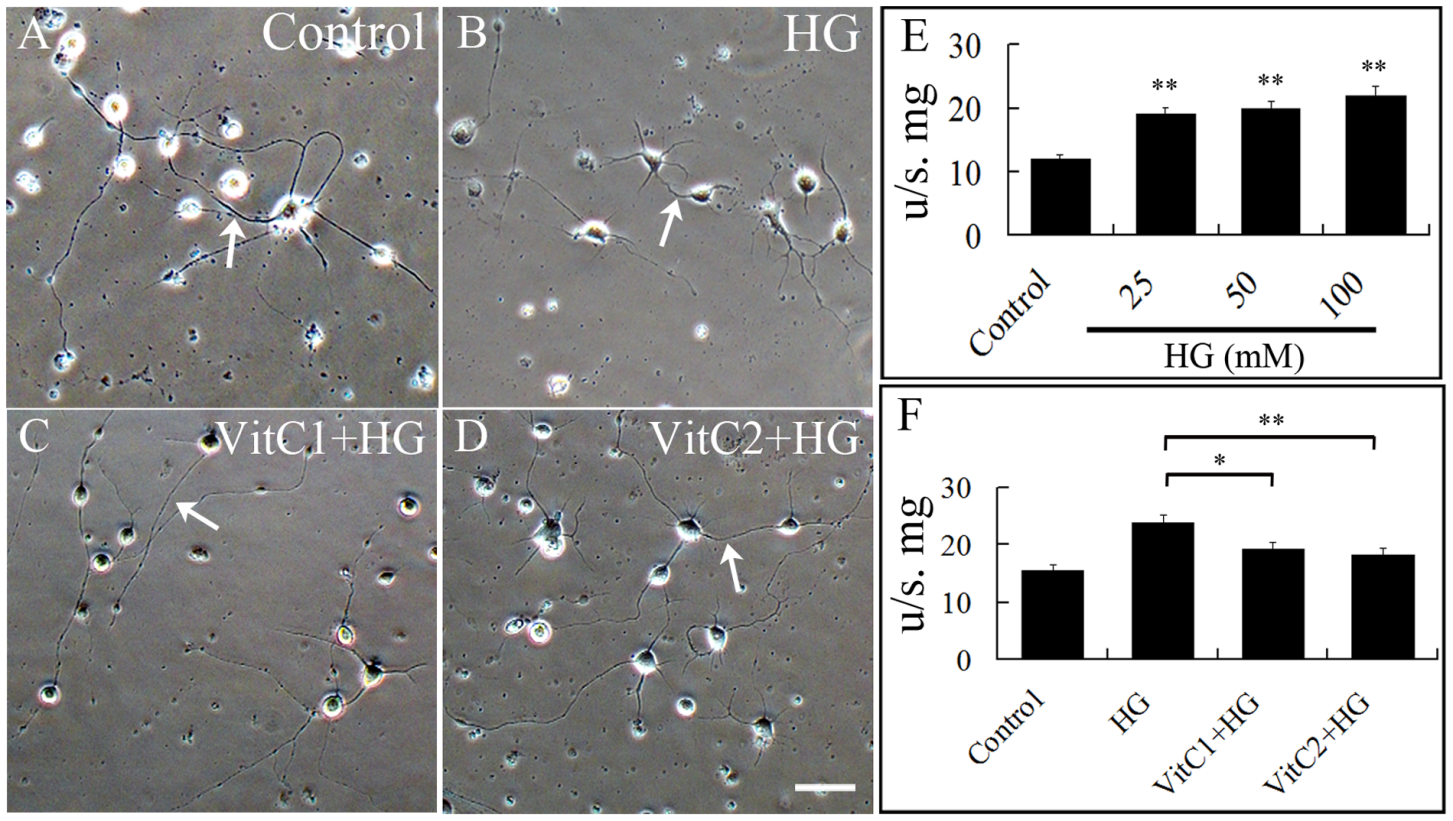
High glucose generated excess ROS and impaired neurodevelopment. **A–D:** Morphology of neurons in control, high glucose (HG) and HG+vitamin C treated cultures. HG reduced the number and length of neurites protruding from the neurons (B). However, addition of vitamin C rescued the harmful effects of HG on the cultured neurons (C and D). **E:** Bar chart showing ROS significantly increased in dose-dependent manner as neurons are exposed to different concentrations of glucose (**p<0.01). **F:** Bar chart showing that the excess ROS generated by the cultured neurons in the presence of high glucose could be inhibited by vitamin C. There was significant difference in ROS generation between high glucose (HG) group and HG+vitamin C groups (*p<0.05, **p<0.01, compared with the HG group). Abbreviations: HG (25 mM high glucose); VitC1 (0.1 mM vitamin C); VitC2 (0.5 mM vitamin C). Scale bar = 50 µm.

We investigated whether excess ROS production played a harmful role on neurons during high glucose exposure. We employed vitamin C as an antioxidant to reduce the excess ROS generated in the experimental cultures (Figs. 7A–D). We first showed that the level of ROS increased as the concentration of glucose was increased (p<0.01, Fig. 7E). There was a mark difference in ROS levels between neuron cultures treated with high glucose and culture treated with high glucose+vitamin C (Fig. 7F), suggesting that vitamin C could mop up the excess ROS produced. Neurons cultured in the presence of 25 mM high glucose was morphologically different from neurons treated with the same concentration glucose but with vitamin C also added (Figs. 7A–D). The neurites of neurons under high glucose were markedly shorter than the neurites of neurons in control cultures (Figs. 7A and B). However, the high glucose-induced dysplasia neurons were rescued following addition of vitamin C (Figs. 7C and D).

## Discussion

Using quail embryos as a model, we investigated the effects of high glucose on embryonic development, especially on neurogenesis. It has been reported that excess glucose increased the risk of macrosomia developing in infants of women with diabetes during pregnancy [22]. This is consistent with the findings in our study that the weight of quail embryos treated with a high dose of glucose was significantly heavier than control embryos. Early placental function with up-regulation of placental glucose transporters may be a factor. Since the glycogen increments in diabetes are predominantly located around fetoplacental vessels, in conditions of fetal glucose levels exceeding the demand for sustaining fetal growth and metabolism, glucose can be stored in the liver and other fetal tissues. Fetal macrosomia would then occur only when fetal hyperglycemia exceeds the placental capacity to store excess fetal glucose [23]. There may be direct genetic factors affecting birth weight. It may be that the presently improved blood glucose control, although still not perfect, has resulted in a reduction in maternal diabetic microangiopathy [24].

Normal neurodevelopment consists of a cascade of key steps. Firstly, the neural folds elevate from the neural plate and fuse together to form the neural tube. The cranial neural tube will become the forebrain, midbrain and hindbrain while rest of the neural tube forms the spinal cord. Secondly, the neurons divide rapidly in the immature head and trunk neural tube. Thirdly, the nerve cells migrate out of neural tube at the distinct level toward their destination [25]. Therefore, if the neural tube failed to properly close for various reasons, it would lead to NTDs. We have demonstrated that NTDs would occur at the trunk level in the presence of high glucose. In addition, the NTDs rate increased in a high glucose dose-dependent manner. It has been reported that Pax7 mutant mice displayed defective neural tube closure and NCC migration [21], [26]. In the quail embryo, NCCs bilaterally migrate out of the dorsal neural tube at stage of HH10–11. The neural tube must close properly for the NCCs to development [27]. In this study, we have established that the migration of Pax7^+^/HNK-1^+^ NCCs in presence of high glucose was impaired and this may have been attributed to the abnormal closure of the neural tube.

NCCs are the precursors of the sympathetic ganglia. They emigrate from the neural tube and migrate toward the dorsal aorta and then move through the sclerotome to form sympathetic neurons or become stalled within the sclerotome to form the sensory ganglia [28], which would be part of the dorsal root. Hence, defects in NCCs development and migration would lead to abnormal development of the dorsal root ganglia. We have demonstrated that there were significantly fewer NF^+^ dorsal root ganglion cells in high glucose-treated embryos than control embryos. Since NCCs contribute to the dorsal root ganglia, we believe that impediment of NCCs migration, because of the high glucose, lead to the dorsal root ganglia dysplasia. In addition, sensory axons from the dorsal root ganglia also grow into the developing limbs in response to diffusible factors (since ectopic limb grafts also become innervated). The axons of the motor neurons in limb bud are derived from the neural tube [29]. Moreover, PNS axons of sensory nerve ganglia are also invade the developing limbs [30]. In this context, the impaired development of motor and sensory nerves that we observed in the developing limb bud under high glucose may be attributed NTDs and defects in the dorsal root ganglia.

Diabetes cause increased oxidative stress in various embryonic tissues, as evidenced by increased levels of oxidized DNA, proteins, and lipids. Oxidative stress dose not only damage the integrity of these molecules but also triggers a series of cellular response that includes the activation of protein kinase C and JNK stress-associated kinases [31]. Accordingly, our findings suggest that excess ROS generated by neurons following high glucose exposure directly inhibited the neurons’ development. The idea that excess ROS affects NCCs development in diabetic pregnancies is supported in the literatures [32], [33]. This suggests that the malformations which developed in the neural tube, dorsal root ganglia and nerves in the limb buds were the results of impaired NCCs development caused by excess ROS in presence of high glucose. It has been reported that vitamin C supplements added to the diet of pregnant diabetic rats could reduced the rate of embryo malformations and fetal resorption [34]. In another word, vitamin C could act as an antioxidant to neutralize excess ROS produced in embryonic tissues. Our study supports this idea because we demonstrated that adding vitamin C to our cultured neurons could inhibit the damaging effects of high glucose on the neurons. Moreover, since we showed that vitamin C decreases ROS generation, it implies that vitamin C exerted its effect by inhibiting ROS rather than directly protecting the cells.

## Materials and Methods

### Quail Embryos

The quail employed in this study belongs to phasianidae, Coturnix Bonnaterre, and lives in Guangdong province of China. The fertilized quail eggs were obtained from the Avian Farm of the South China Agriculture University. The eggs were incubated in a humidified incubator (Yiheng Instrument, Shanghai, China) at 38°C and 70% humidity. After 36 hour incubation, the quail embryos were treated with different concentrations of glucose or mannitol (control). Briefly, approximately 100 µl of various concentrations of glucose or mannitol were injected into the egg via a small hole made at the blunt-end of the egg. The high glucose or mannitol could be directly drops on the embryos utilizing this experimental strategy (Figure S1). The treated embryos were then incubated for a further 48–96 hours before they were fixed with 4% paraformaldehyde (PFA) for analysis.

### Whole-mount Embryo Immunostaining

Quail embryos were harvested after 3.5–5.5 day incubation and fixed in 4% PFA overnight at 4°C. Whole-mount embryo immunostaining was performed using the following antibodies: neurofilament (NF; 1∶500, Invitrogen, USA), Pax7 (1∶100, DSHB, USA) and HNK-1 (1∶500, DSHB, USA). Briefly, the fixed quail embryos were incubated with NF, Pax7 or HNK-1 antibody at 4°C overnight on a shaker. After extensive rinsing in PBS, the embryos were incubated with alexa fluor 555 anti-mouse IgG secondary antibody (1∶1000, Invitrogen, USA) at 4°C overnight on a shaker. All the embryos were later counterstained with DAPI (1∶1000, Invitrogen) at room temperature for 1 hour.

### Silver Staining

The paraffin sections were de-waxed in xylene and hydrated in a graded series of ethanol (100% ethanol for 5 min, 95% ethanol for 5 min, 80% ethanol for 5 min, 70% ethanol for 5 min, distilled water for 10 min). Silver nitrate (20%) solution was heated at 60°C for 15 min, and then sections were placed into the solution for 15 min and rinsed with tap water. Silver ammonia staining was then carried out for 5–30 min and the sections were rinsed with tap water 3 times. Sodium thiosulfate (5%) staining was performed for 2 min, washed in tap water and then mounted with neutral gum.

### Primary Neuron Culture

Primary neuron culture was prepared from the brain of 11-day old quail embryos, according to modified procedures as previously described [35]. Briefly, the brain was dissected out from the embryos and enzymatically dissociated into single cells. The cells were plated in Neurobasal media supplemented with 2% B27 at a density of 3.6×10^5^ cells/ml. The primary neuronal cultures were maintained in an incubator at 37°C and 5% CO_2_. Half of the medium in each culture was changed every 72 hours. The cultured neurons were treated with 25–100 mM of glucose or mannitol (control) for 24 hours.

### Primary Culture of NCCs

NCCs were prepared from the neural tube (at trunk level) of quail embryos, according methods previously described [36]. Briefly, fertilized quail eggs were incubated until the 10–13 somite stage (HH 10–11) [37]. The neural tubes were dissected from the trunk of the embryos and explanted into 6-well plates in DMEM (1500 µl/well) for 24 hours at 37°C and 5% CO_2_. A mass of NCCs migrated from the neural tubes after 4-hour incubation and then different concentrations of glucose or mannitol solutions were introduced into the cultures for 24 hours.

### Immunostaining in Primary Neuronal Culture

After cells were exposed to high concentrations of glucose for 24 hours *in vitro*, they were fixed for 30 minutes in 4% PFA in PBS, rinsed and treated with normal goat serum. The specimens were then incubated in primary Tuj1 antibody (1∶200, Invitrogen, USA) and then secondary goat anti-mouse IgG 555 (1∶1000, Invitrogen, USA). Lastly the specimens were counterstained with DAPI dye (1∶1000, Invitrogen, USA). At least 3 replicates were analyzed for each treatment and photographed under an inverted fluorescent microscope.

### Photography

Following immunohistochemistry, the whole mount embryos were photographed using a stereo-fluorescent microscope (Olympus MVX10) and associated Olympus software package Image-Pro Plus 7.0. The embryos were sectioned into 15 µm thick slices using a cryostat microtome (Leica CM1900), and then photographed using an epi-fluorescent microscope (Olympus LX51, Leica DM 4000B) with a CN4000 FISH Olympus software package.

### Data Analysis

Data analyses and creation of charts were performed using a Graphpad Prism 5 software package (Graphpad Software, CA, USA). The results were presented as the mean value (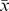
±SE). All data were analyzed using ANOVA, which was employed to test the difference among experimental groups. P<0.05 was considered to be significance.

## Supporting Information

Figure S1 **The strategy for administering high glucose or mannitol to early quail embryos in vivo.** Schematic drawing of high glucose or mannitol introduced into early quail embryos in vivo. The fertilized eggs pre-incubated for 1.5 days were holed and treated with high glucose or mannitol, and then sealed and continually incubated for a further 2 days or 4 days.
(TIF)
