# Supplementary figures and images for: The Negative Influence of High-Glucose Ambience on Neurogenesis in Developing Quail Embryos
Source: PLoS One. 2013 Jun 20;8(6):e66646. doi: 10.1371/journal.pone.0066646 (PMC3688607; doi:10.1371/journal.pone.0066646)

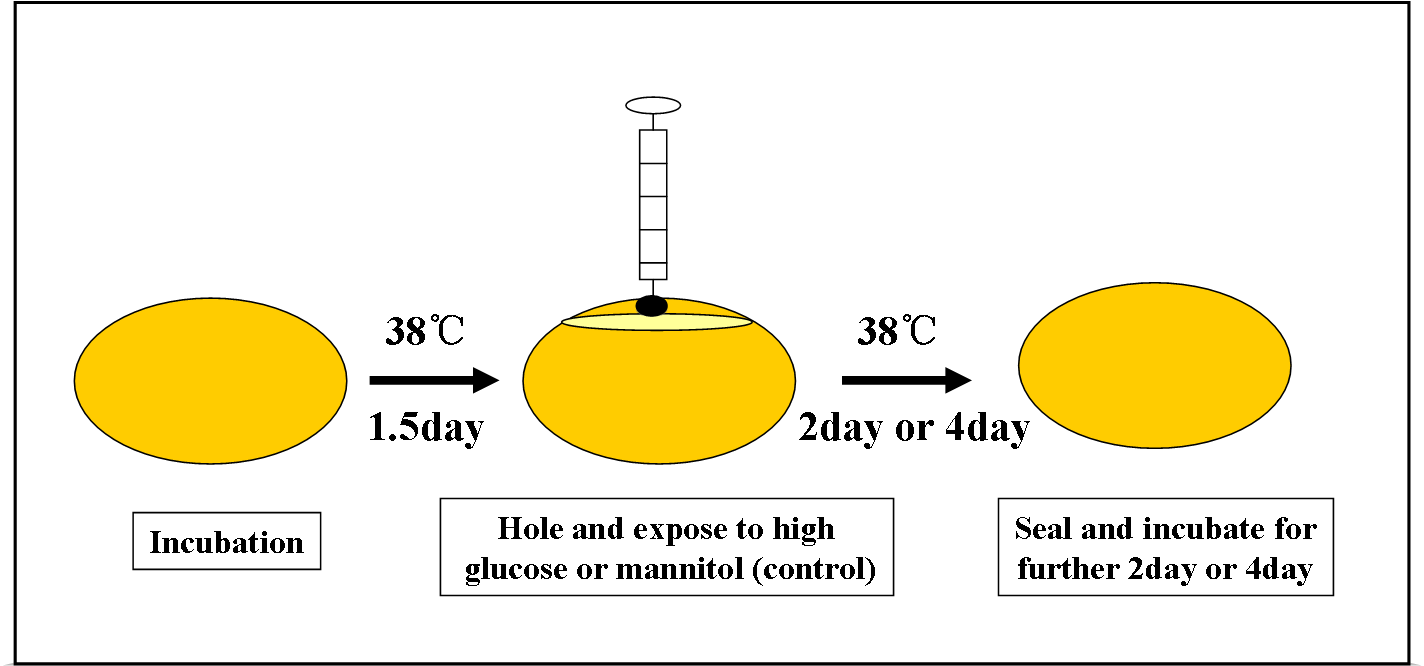

Supplement: Figure S1 — The strategy for administering high glucose or mannitol to early quail embryos in vivo. Schematic drawing of high glucose or mannitol introduced into early quail embryos in vivo. The fertilized eggs pre-incubated for 1.5 days were holed and treated with high glucose or mannitol, and then sealed and continually incubated for a further 2 days or 4 days. (TIF) [file pone.0066646.s001.tif]
